# Structure and Heat Transfer in Zircaloy-4 Treated at High Temperatures

**DOI:** 10.3390/ma14164494

**Published:** 2021-08-10

**Authors:** Mărioara Abrudeanu, Maria Magdalena Dicu, Maria Minodora Pasăre

**Affiliations:** 1 Department of Materials Science and Engineering, Technical Science Academy of Romania, 030167 Bucharest, Romania; 2 Department of Manufacturing and Industrial Management, University of Pitesti, 110040 Pitesti, Romania; ela_magda@yahoo.com; 3 Faculty of Engineering, University Constantin Brancusi, 210163 Targu Jiu, Romania; minodora_pasare@yahoo.com

**Keywords:** zircaloy, high temperatures, corrosion, structural transformations, microhardness, thermal diffusivity

## Abstract

Zircaloy-4 has an important role in the construction of generation III nuclear reactors. An important application is the fuel element sheath, which must have excellent corrosion resistance in the working environment, adequate mechanical characteristics and very good heat transfer properties from the combustible element to the coolant. The corrosion processes at high temperatures, the accidents that lead to significant increases in temperature and the structural transformations associated with them affect the heat transfer process. The paper presents research on the influence of high temperatures on the microstructure and thermal diffusivity of the zy-4 alloy. The samples were treated in air, at temperatures between 850 and 1050 °C for 60 min. The corrosion layers were characterized microstructurally and chemically. Furthermore, the transformations produced in the base material under the corrosion layer were analyzed. The values of thermal diffusivity were determined and correlated with the structural transformations. Considering the state of research on the materials appropriate to be used for new generation reactors, the current importance of third-generation reactors for energy systems and the fact that they will operate in the coming years, we consider that the study offers useful outcomes in the field of nuclear energy.

## 1. Introduction

An important part of the electricity used today in the world is supplied by nuclear power plants [1,2]. “Safety first” has always been the motto and essential concern of nuclear energy, and yet there have been accidents around the world that were thought to never occur. Nuclear power, through its capacity for self-improvement, has continuously improved safety performance [3,4,5]. An important factor both for the generation III reactors in operation today—which are currently intended to have their life-cycle extended—and for the new generation reactors is the heat transfer through the fuel element sheath [6]. In the construction of CANDU reactors, the fuel element sheath is made of zircaloy-4 [7,8,9,10,11,12]. In a normal temperature regime, 350 °C, it forms on the surface a thin, compact and adherent oxide layer, with a protective character [13,14,15,16]. The accidental increase of the temperature towards high values determines the increase of the thickness and the modification of the structure of the corrosion layers, as well as important changes in the structure and properties of the alloy [17,18,19,20,21,22].

The objectives of this experimental research were to determine the influence of the oxide layers formed on the sheath and the high temperatures on the heat transfer properties of the Zircaloy-4 sheath.

## 2. Materials and Methods

**Figure 1:**
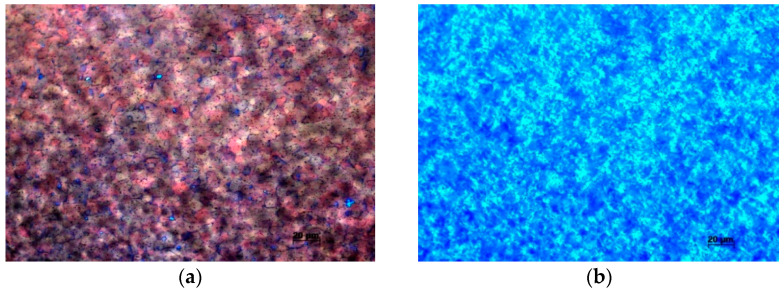
Metallographic structure of Zircaloy-4 (bar) (**a**). longitudinal section; (**b**). cross section.

Cylindrical samples made of Zy-4 (Sn—1.31%, Fe—0.2% and Vr—0.11% C), bar, with a diameter of 10 mm were used in the experimental study. The micro-hardness in the cross section is 228 V and in the longitudinal section, it was 254 HV. Vickers microdurity measurement was performed with the O.P.L. France automatic cycle microdurimeter with the measurement accuracy of ± 10%. The grain size of the Zy-4 alloy in its initial state is very small (Figure 1). For zircaloy bars, the grain size measured along any normal line at the sample surface must be less than 30 µm, and the maximum grain size must not exceed 80 µm [23,24].

The microstructural characterization was performed with CARL ZEISS optical microscope and TESCAN Vega II LMU electron microscope. The grain size for the initial state was determined according to the ASTM E 112/1988 standard, on mechanically sanded samples on abrasive paper of granulation up to 1200 µm, with a chemical attack in a solution of 45% HNO_3_, 45% H_2_O and 10% HF, followed by oxidation anodized in an electrolyte of 120 mL ethyl alcohol, 20 mL glycerin, 70 mL distilled water, 40 mL lactic acid (85%), 10 mL phosphoric acid (85%) and 4 g citric acid. Washing was performed with warm water and ethyl alcohol, and drying in hot air.

The estimation of the grain size was carried out manually by the Heyn interception method to determine the average grain size and the confidence limit. The results obtained were consistent with those in the literature and the standard.

For the large grain sizes, characteristic of oxidized samples at high temperatures, at least 5 metallographic images were used on which test lines were drawn, hence determining the number of interceptions with the studied surfaces.

The samples were treated in air, in the constant temperature zone of the oven, at temperatures between 850 and 1050 °C. They were characterized by measurements of the thickness of the corrosion layers, determinations of thermal diffusivity, optical and electron microscopy, determinations of chemical composition in the section at the electron microwave probe, measurements of grain size and microhardness.

The thermal diffusivity was determined by the “flash” method, in the first stage for the samples with the corrosion layers on both sides [25,26,27,28,29,30]. In view of the results obtained, in order to better highlight the role of the layer thickness, in relation to that of the microstructure, additional preparations were made through the removal of oxide layers by polishing to metallic luster for both sides of the sample, followed by diffusivity determinations.

The value of thermal diffusivity was calculated as the average of the values determined on the two faces, each of them being the average of 10 measurements. After determining the thermal diffusivity, the samples were examined microscopically in section, and microhardness measurements were performed and analyzed by electronic microsound. Finally, in order to highlight the role of structural transformations, we determined the continuous variation of the Zy-4 alloy diffusivity at heating and cooling without maintenance at 1050 °C and with a maintenance of 30 min.

## 3. Experimental Results

**Figure 2:**
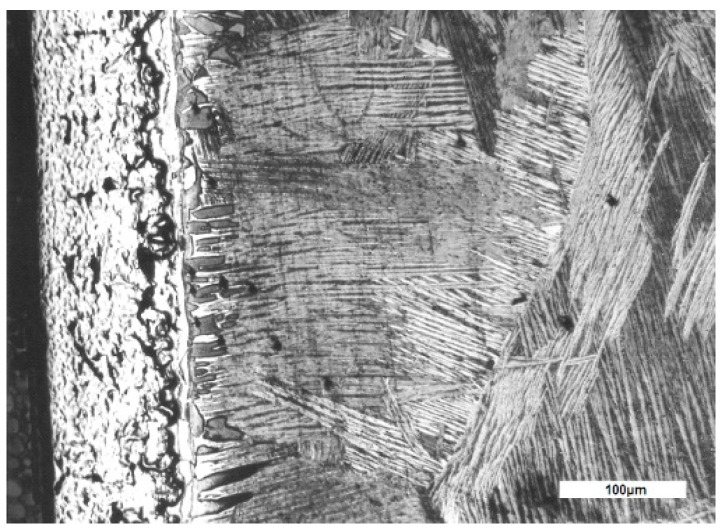
Microstructure in section of the sample treated 60 min at 1050 °C, MO.

Optical microscopic analysis in the section of the samples highlighted the types of structures formed, in relation to the surface and the oxide–metal interface: the oxide layer, an area of stabilized solid solution α that develops under the oxide layer by dissolving oxygen in the metal and the structure of the core (Figure 2).

**Figure 3:**
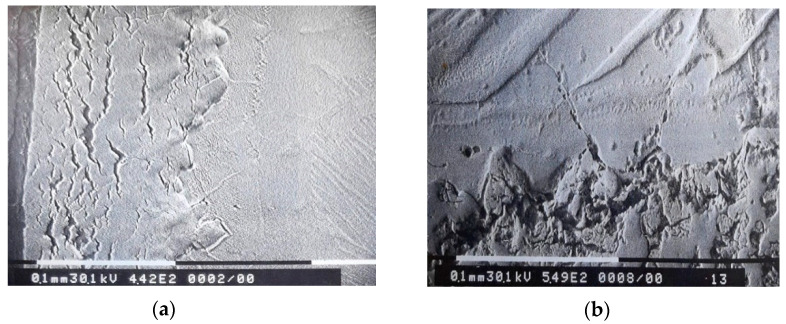
MEB microstructure of corrosion layers. Degradation processes in the oxide layers and at the oxide–metal interface at (**a**) 1000 °C, (**b**) 1050 °C.

The corrosion layer, formed mainly from oxides, shows deterioration processes in the form of pores and cracks. Layer cracks are formed prevailingly in parallel to the surface of the sample (Figure 2 and Figure 3a).

**Figure 4:**
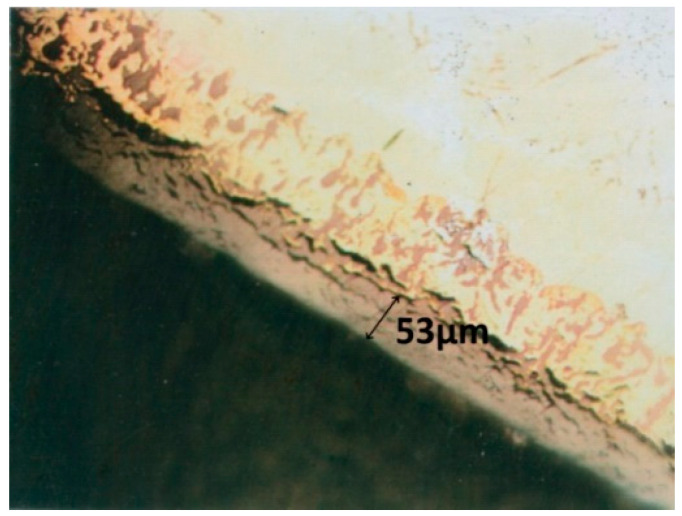
The area with oxynitrides in the corrosion layer after treatment at 951 °C.

The corrosion layer displays nitrogen-rich, yellowish areas near the interface with the metal (Figure 4).

Cracks appear at the oxide–metal interface that propagate towards the core of the metallic mass (Figure 3b).

The solid solution layer α develops under the oxide layer with a columnar growth, preferentially in perpendicular directions at the oxide–metal interface (Figure 2).

The core of the sample consists of grains of solid solution β in the form of needles and platelets whose orientation differs from one grain to another.

**Figure 5:**
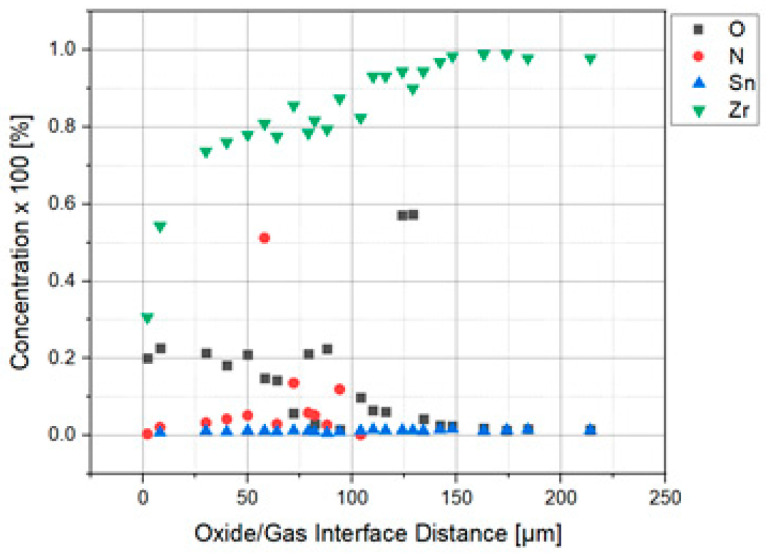
Evolution of the chemical composition on the isothermal oxidized sample section at 1050 °C for one hour.

The analysis in the electronic microbe probe highlighted the distribution of the elements in the section. The variation of the oxygen content is well correlated with the thickness of the oxide layer, with the formation of the solid solution layer α stabilized by dissolving the oxygen under the layer and shows the continuous decrease of the oxygen concentration in the core with the structure in needles and platelets. The presence of nitrogen is marked near the oxide–metal interface (Figure 5).

**Figure 6:**
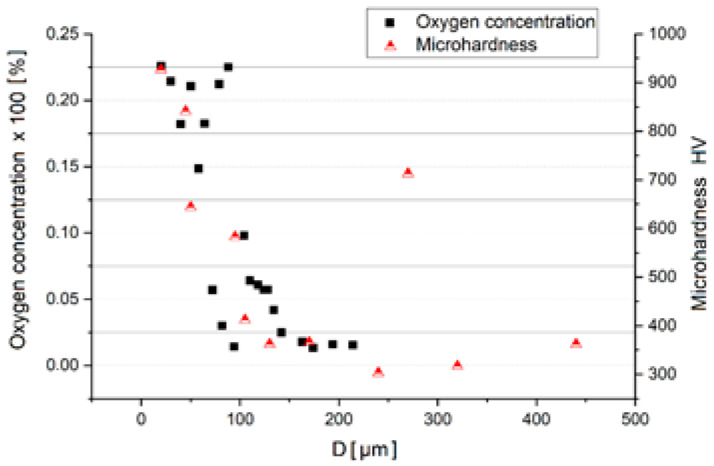
Correlation between oxygen content and microhardness values for isothermal oxidized sample 60 min at 1050 °C.

The determinations of microhardness in section, correlating with the oxygen content of the sample, highlighted the effects of dissolving the oxygen to stabilize an area with α phase and structural hardening (Figure 6).

**Figure 7:**
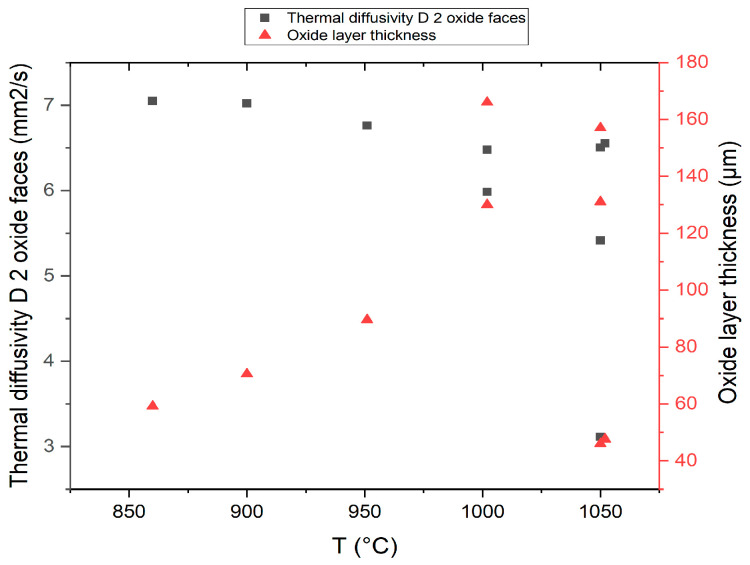
The influence of temperature on the thickness of the corrosion layers and on the thermal diffusivity.

The thermal diffusivity values of the samples with oxide layers on both sides show a continuous decrease with the isothermal oxidation temperature (Figure 7). At high temperatures, it was found that the diffusivity is not always well correlated with the determined thickness of the oxide layer, which may be due to differences in thickness resulting from the detachment of a part of the layer.

**Figure 8:**
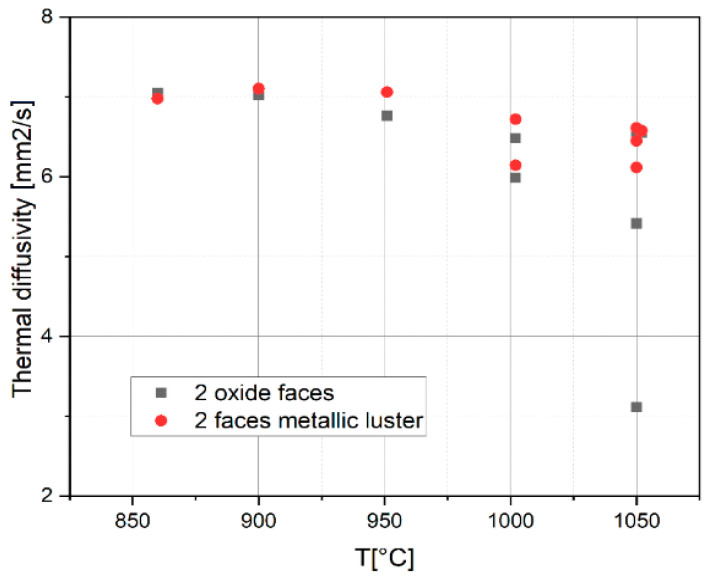
Thermal diffusivity values depending on the treatment temperature for the samples with both sides covered with oxide and with faces polished to metallic luster.

In order to establish the effect of the presence of corrosion layers, we determined the thermal diffusivity of the samples without the corrosion layer, with both sides polished to metallic luster (Figure 8). The values of the thermal diffusivity determined after the complete removal of the corrosion layer on both sides are higher than those corresponding to the samples with oxide layers, but they decrease when the treatment temperature increases, which shows the influence of microstructural transformations.

**Figure 9:**
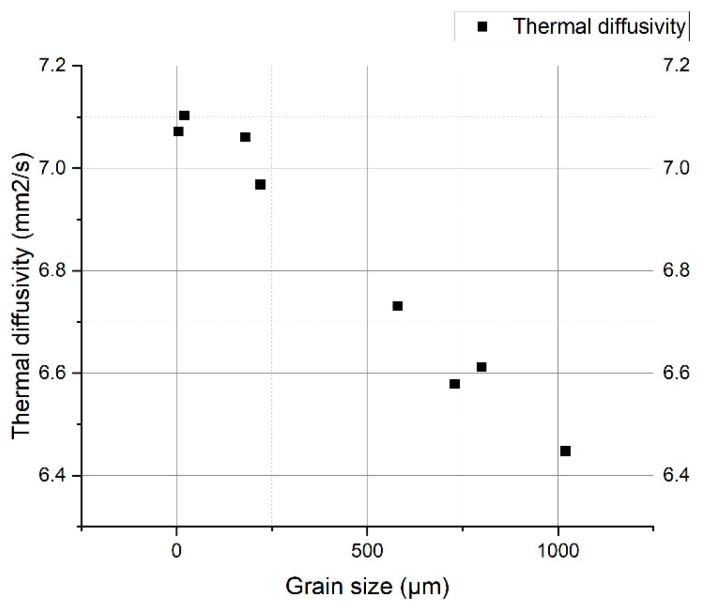
Variation of thermal diffusivity with grain size of samples without corrosion layers.

The correlation of the thermal diffusivity values with the values of the grain size of the core is shown in Figure 9.

**Figure 10:**
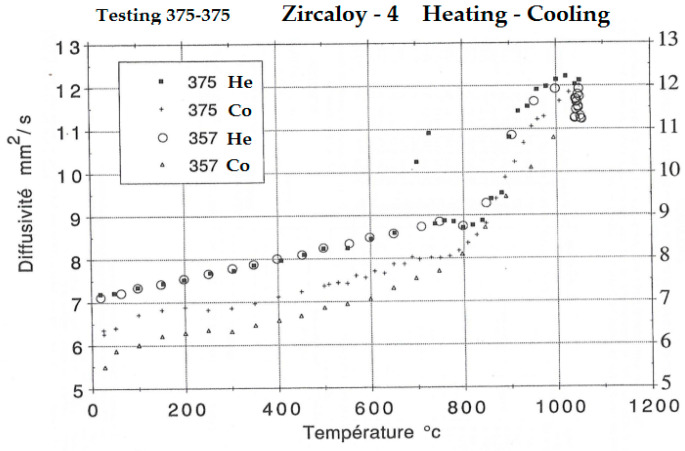
Variation of the thermal diffusivity of the sample during a treatment cycle consisting of heating at 1050 °C and cooling, with maintenance and without maintenance of 30 min at 1050 °C.

In order to highlight the role of the transformations that occur when the alloy is heated to 1050 °C and for cooling, continuous diffusivity determinations were made for a heating = cooling cycle, without maintenance at 1050 °C and a heating–cooling cycle with maintenance of 30 min at 1050 °C (Figure 10).

Both curves showed that, without the presence of corrosion layers, the curves of the thermal diffusivity values determined at cooling are below those at heating. Maintaining a high temperature causes a significant decrease in diffusivity.

## 4. Interpretation of Results

**Figure 11:**
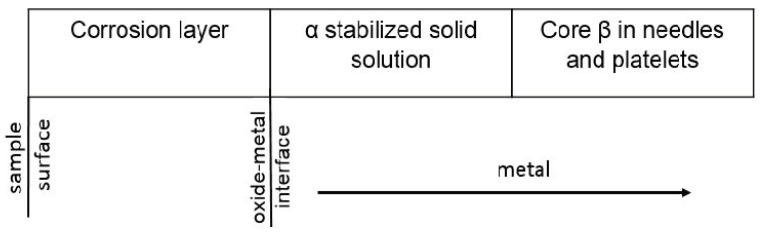
Schematic representation of the structural constituents on the sample section in relation to the oxide–metal surface and interface.

At the microstructural level, research has shown that the formation and increase of corrosion layers, mass transfer processes in the layer, through the oxide–metal and metal interface, associated with the transformations caused by high temperatures, lead to the formation of the following types of structures on the thickness of the sample:

- a corrosion layer with an oxide zone in the upper part, which presents degradation defects in the form of pores and cracks and with nitrogen-rich precipitates located near the oxide–metal interface
- a layer of solid solution α stabilized by dissolving oxygen in the metal, under the oxide layer, with a columnar development towards the core and
- the core with large grains of solid solution β, in needles and platelets (Figure 1 and Figure 11).

The oxide–metal interface has cracks that penetrate the metal mass and can be diffusion paths for oxygen. Nitrogen-rich areas are more compact and could be barriers to oxygen diffusion.

The analysis of the chemical composition in the section highlights the variation of the oxygen content in the oxide layer and in the metal under the oxide layer.

The variation profile of the microhardness is well correlated with the oxygen concentration in the sample, marking the stabilizing effect of the alpha solid solution and the hardening of the alloy.

Thermal diffusivity measurements for oxidized samples have a descending profile relative to the increase of treatment temperature. After removing the oxide layer at metallic luster on both sides, the diffusivity values keep a decreasing temperature profile. The correlation of the diffusivity values with the grain size in the beta phase shows an accentuated decrease of the thermal diffusivity with the increase of the grain size.

Continuous variation curves of thermal diffusivity for the complete heating–cooling cycle at 1050 °C show the effect of structural transformations and the irreversibility of the cooling phenomenon, the cooling diffusivity curve being constantly below that of heating. Maintaining the temperature at 1050 °C has an additional effect by increasing the grain size.

## 5. Conclusions

The present research highlighted the structural areas formed on the Zy-4 alloy during isothermal corrosion in air at high temperatures. The chemical composition in the section was determined and correlated with the present structural areas.

The microhardness measurements in the section give the effect of oxygen on structural hardening. The hardness variation curve is well correlated with the oxygen concentration variation curve.

The values of thermal diffusivity decrease with the increase of the isothermal oxidation temperature and with the increase of the thickness of the oxide layer. After the removal of the oxide layer, the thermal diffusivity curves are located above the curve with oxide layers, following a decreasing temperature rate (allure), which leads us to the idea that there is a main, structural cause, proven by the hardness curve with grain size and the diffusivity variation curves in the heating-cooling treatment cycle.

The research highlights the important effect of structural transformations at high temperatures on thermal diffusivity.

## Data Availability

The data presented in this study are available on request from the corresponding author.
